# Response of the gut microbiome and metabolome to dietary fiber in healthy dogs

**DOI:** 10.1128/msystems.00452-24

**Published:** 2024-12-23

**Authors:** Amrisha Bhosle, Matthew I. Jackson, Aaron M. Walsh, Eric A. Franzosa, Dayakar V. Badri, Curtis Huttenhower

**Affiliations:** 1 Infectious Disease and Microbiome Program, Broad Institute of MIT and Harvard, Cambridge, Massachusetts, USA; 2 Department of Biostatistics, Harvard T. H. Chan School of Public Health, Boston, Massachusetts, USA; 3 Harvard Chan Microbiome in Public Health Center, Harvard T. H. Chan School of Public Health, Boston, Massachusetts, USA; 4 Hill’s Pet Nutrition Inc., Topeka, Kansas, USA; 5 Department of Immunology and Infectious Diseases, Harvard T. H. Chan School of Public Health, Boston, Massachusetts, USA; University of California Irvine, Irvine, California, USA

**Keywords:** canine, dietary fiber, metabolome, gut microbiome, personalized nutrition

## Abstract

Dietary fiber confers multiple health benefits originating from the expansion of beneficial gut microbial activity. However, very few studies have established the metabolic consequences of interactions among specific fibers, microbiome composition, and function in either human or representative animal models. In a study design reflective of realistic population dietary variation, fecal metagenomic and metabolomic profiles were analyzed from healthy dogs fed 12 test foods containing different fiber sources and quantities (5–13% as-fed basis). Taxa and functions were identified whose abundances were associated either with overall fiber intake or with specific fiber compositions. Fourteen microbial species were significantly enriched in response to ≥1 specific fiber source; enrichment of fiber-derived metabolites was more pronounced in response to these fiber sources. Positively associated fecal metabolites, including short-chain fatty acids, acylglycerols, fiber bound sugars, and polyphenols, co-occurred with microbes enriched in specific food groups. Critically, the specific metabolite pools responsive to differential fiber intake were dependent on differences both in individual microbial community membership and in overall ecological configuration. This helps to explain, for the first time, differences in microbiome-diet associations observed in companion animal epidemiology. Thus, our study corroborates findings in human cohorts and reinforces the role of personalized microbiomes even in seemingly phenotypically homogeneous subjects.

**IMPORTANCE:**

Consumption of dietary fiber changes the composition of the gut microbiome and, to a larger extent, the associated metabolites. Production of health-relevant metabolites such as short-chain fatty acids from fiber depends both on the consumption of a specific fiber and on the enrichment of beneficial metabolite-producing species in response to it. Even in a seemingly homogeneous population, the benefit received from fiber consumption is personalized and emphasizes specific fiber-microbe-host interactions. These observations are relevant for both population-wide and personalized nutrition applications.

## INTRODUCTION

The gut microbiome plays an important role in host health-related biological processes such as energy harvest, immunity, and detoxification (1–3). Nutrients and compounds from food can directly influence the microbiome, and gut microbial metabolism of these compounds can in turn influence the host (4). These effects are most pronounced with homogeneous diets, which can occur in human populations (e.g., traditional hunter-gatherers [5]) or in laboratory or free-living animals (6–8). Companion animals thus provide a particularly relevant context in which to study food-microbiome interactions since their diets are typically more monotonous and better controlled than those of humans, but they occupy human-like non-laboratory environments, and the corresponding food-microbiome relationships have the potential to be leveraged to improve both human and animal health (9, 10).

This context is especially true with respect to interactions between specific gut microbes and dietary fiber sources, which are extensive in both humans and companion animals (11–13). Dietary fiber interventions carried out in humans and dogs resulted in alterations to microbiome structure and metabolism (11, 12, 14–17), reflective of their surprisingly analogous microbial ecology (composed of the same dominant phyla [18]) and genetic material (19). Moreover, dogs share similar dietary, environmental, and lifestyle habits with their human counterparts (20). In all mammalian hosts, short-chain fatty acids (SCFAs) are among the major products of fiber fermentation in the colon, where they participate in intestinal homeostasis and immune regulation (21). Additionally, specific fiber-containing ingredients entrap bioactive plant secondary metabolites such as polyphenols that are not bioavailable to the host until liberated by resident microbes (22). These polyphenols and the products of their catabolism by gut microbes additionally affect gastrointestinal physiology (14). Certain fibers also improve the bioavailability of minerals (14, 23, 24) and affect the microbial metabolism of residual dietary carbohydrates and proteins (11, 14). However, differentiating the effects of individual fibers in complex human diets is challenging, and insights about the resulting interactions among fiber, microbes, and host phenotypes have been limited to a small number of supplementation trials (25–27).

Beyond fiber as a major dietary modulator of the microbiome in most animals, several studies in both humans and dogs have examined the broader relationship among food variables such as macronutrients, processing, ingredient sources, and the microbiome (11, 28–32). The most targeted dietary interventions studied with respect to the human microbiome include extreme shifts between fiber-rich and poor diets, which resulted in modest changes to microbial composition but highly significant alterations in SCFA production and associated transcripts (29). Similarly, the Food and Resulting Microbial Metabolites study in humans contrasted omnivore, vegan, and exclusive enteral nutrition (EEN) diets differing (among other ways) in fiber content, observing that the lack of fiber in EEN was arguably the greatest impediment in the recovery of the microbiome from antibiotic treatment (11). In dogs, a study comparing the effects of extruded diets found that the gut microbiomes in response to commercial diets containing both plant and animal protein versus animal protein-free diets were similar (31), indicating that coarse macronutrient similarity (and not the source of the macronutrients) is potentially able to maintain comparable microbiomes, in agreement with human studies. Conversely, microbiomes were different in dogs fed a high protein food with less fiber versus extruded foods (32) as well as in foods with different sources of animal proteins (raw beef vs. raw chicken) (30). These details reflect the biochemical niche specialization of gut microbes, but they are extremely challenging to study in human populations with highly individualized microbiomes and variable diets. Apropos, a study was carried out to investigate gut microbial and metabolomic responses to various dietary fiber sources and quantities using a canine colony population. This design allowed the association of specific microbial responses with different classes of carbohydrates (particularly starch vs. fiber), as well as with the accompanying changes in colonic metabolomic profiles stemming from intervention with polyphenol-containing fibers.

## RESULTS

### Design of canine dietary intervention and overview of microbiomes and metabolomes in response to control food

**Fig 1:**
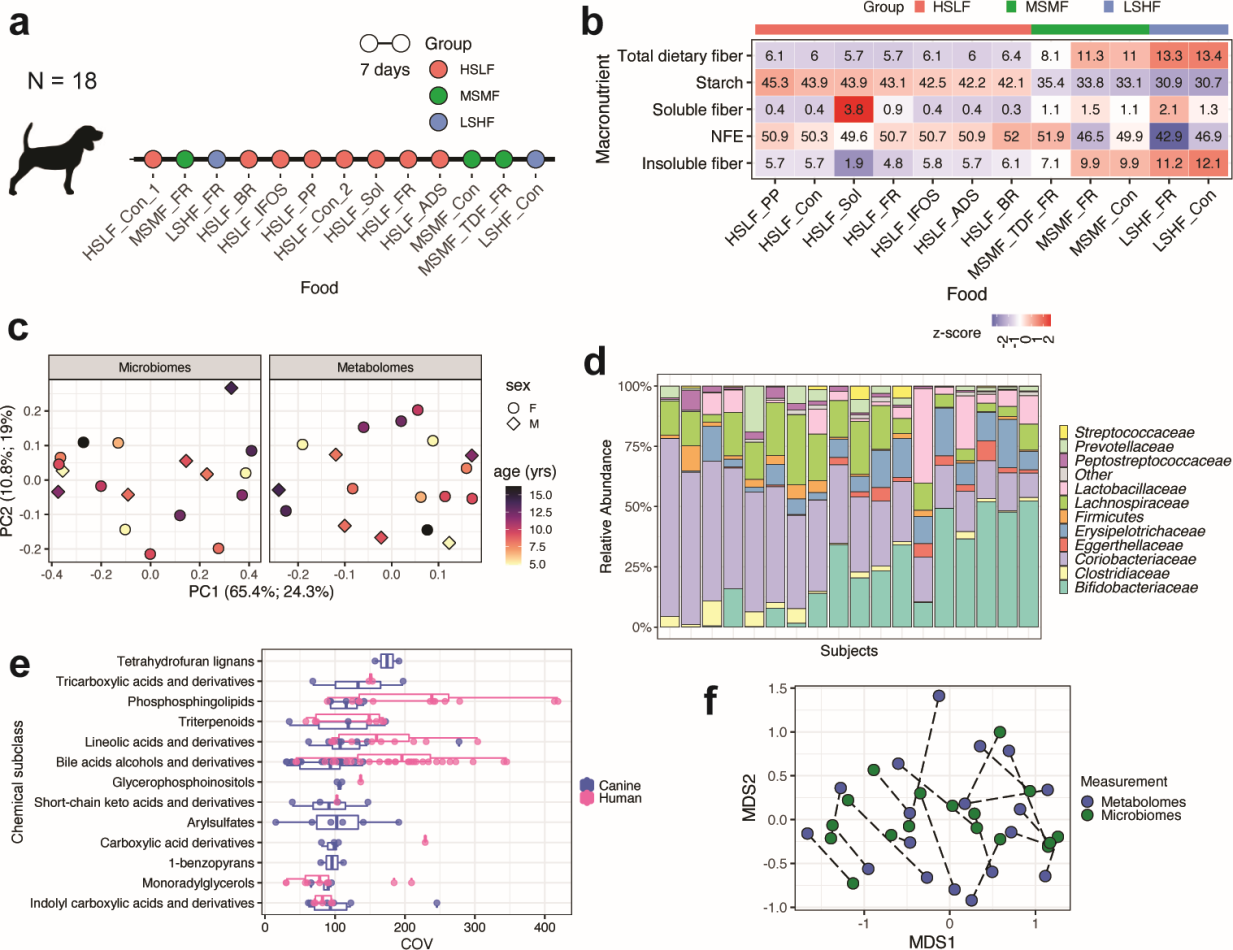
Microbiome and metabolomic profiles in response to starch/fiber food intervention in healthy dogs. (a) Design of the dietary fiber intervention study: 18 dogs were fed 12 foods for a period of 7 days each in a single random order (details in Table S2). Foods are listed in the order they were fed. Fecal samples were collected on the 7th day of each period for metabolomic and metagenomic profiling. Based on fiber and starch content, foods were classified into three groups: HSLF, MSMF, and LSHF. (b) Composition of foods based on fiber types, starch, and NFE. Numbers indicate percentages of macronutrients in each food. Foods are listed according to their starch content. (c) Bray-Curtis principal coordinate analysis of microbiomes and metabolomes following consumption of the control food (HSLF_Con_1). (d) Relative abundances of microbial families in the first sample (HSLF_Con_1) across dogs. Species-level profiles are shown in Fig. S1, and detailed taxonomic profiles (i.e., relative abundances measured using MetaPhlAn) are provided in Table S3. (e) Chemical subclasses in the HSLF_Con_1 fecal metabolomes of dogs and those of healthy humans for comparison from the PRISM cohort (*n* = 34). (f) Procrustes analysis of microbiomes and metabolomes following consumption of HSLF_Con_1. Points from the same dog are connected. ADS, adsorbant; BR, brewers’ rice; Con, control; COV, coefficient of variation; FR, soluble/insoluble fiber ratio; HSLF, high starch, low fiber; IFOS, inulin, fructooligosaccharides; LSHF, low starch, high fiber; MDS, multidimensional scaling; MSMF, medium starch, medium fiber; NFE, non-fermentable energy; PC, principal component; PP, plant protein; Sol, highly soluble fiber; TDF, total dietary fiber.

A total of 18 healthy adult dogs (6 neutered males and 12 spayed females; 15 beagles and 3 mixed breeds), all owned by Hill’s Pet Nutrition Inc., were included in this study (Table S1). The mean age was 9.4 years (range 4.9–16.2 years). All dogs were healthy throughout the study, and no adverse events were reported. Dogs were fed in a non-randomized sequential design for consecutive periods of 7 days each, after which they were moved to the next food without a washout period (Fig. 1a). At initiation (week 1) and midway through the study (week 7), animals received a control food (high starch, low fiber control [HSLF_Con]), representative of commercially available foods (see Materials and Methods).

The remaining 11 test foods were classified into three groups based on their starch and fiber content: 6 HSLF; 3 medium starch, medium fiber (MSMF); and 2 low starch, high fiber (LSHF) (Fig. 1b). The foods contained the same carbohydrate and protein sources as the control food, except one that contained hydrolyzed soy protein (HSLF_plant protein [HSLF_PP]) rather than hydrolyzed chicken as the protein source, and another contained rice as the carbohydrate source (HSLF_brewers’ rice [HSLF_BR]) rather than cornstarch. Foods represented in each group also varied based on their fiber sources (Table S2). The intake of the foods in kcal/metabolic body weight (MBW, where MBW is body weight in kg^0.75^) ranged from a maximum of 114.36 ± 26.13 kcal/MBW for HSLF_Con to a minimum of 92.68 ± 22.23 kcal/MBW) for MSMF_Con (Table S2).

Fecal samples were collected on the last day of each food treatment period for metagenomic and metabolomic profiling. No bias due to age, sex, or breed was observed in the canine microbiomes or metabolomes in response to the control food (Fig. 1c; Fig. S1). Interestingly, phylum Actinobacteria (Bifidobacteriaceae and Coriobacteriaceae families) was dominant in the HSLF_Con_1 microbiomes of dogs in this study, with a mean relative abundance of 59.33%, followed by Firmicutes (36.98%) and Bacteroidetes (3.23%). Fusobacteria and Proteobacteria were of low abundance (Fig. 1d; full taxonomic profiles are in Table S3). Relatedly, *Collinsella intestinalis* and *Bifidobacterium pseudolongum* were the most abundant species (Fig. S1). Overall, the gut microbiomes of dogs included in this study were more similar than those of free-living (33) and facility-housed dogs (34) included in other studies and free-living human subjects from the HMP2 cohort (35) (Fig. S2).

The abundances of a total of 818 post-quality control metabolites were assayed from the same fecal samples. Detected metabolites were chemically diverse, spanning 53 classes and 95 subclasses (Table S4; full metabolomic data are available in Table S5). Several carboxylic acids, fatty acyls, steroids and steroid derivatives, and glycerophospholipids were detected, in contrast to 18 classes represented a single metabolite. Analysis of the coefficient of variation (COV) for each metabolite and their distributions in each subclass showed that metabolite families associated with microbial metabolism in the gut, such as bile acid alcohols and derivatives, phosphosphingolipids, and triterpenoids, were among the subclasses with highest median COVs (Table S4). This indicates the presence of differential microbial metabolic activity in the control food metabolomes and is consistent with observations in the fecal metabolomes of healthy humans (35, 36) (Fig. 1e). Procrustes analysis showed that interindividual variation in the canine fecal metabolome was concordant with variation in the microbiome in response to the control food (*r* = 0.7, *P* < 0.001; Fig. 1f). Thus, despite some variation among animals at baseline, particularly in terms of microbiome composition, dogs with more similar microbiomes also tended to have more similar metabolomes.

### Dietary alterations induce complementary changes in the gut microbiome and fecal metabolome of dogs

**Fig 2:**
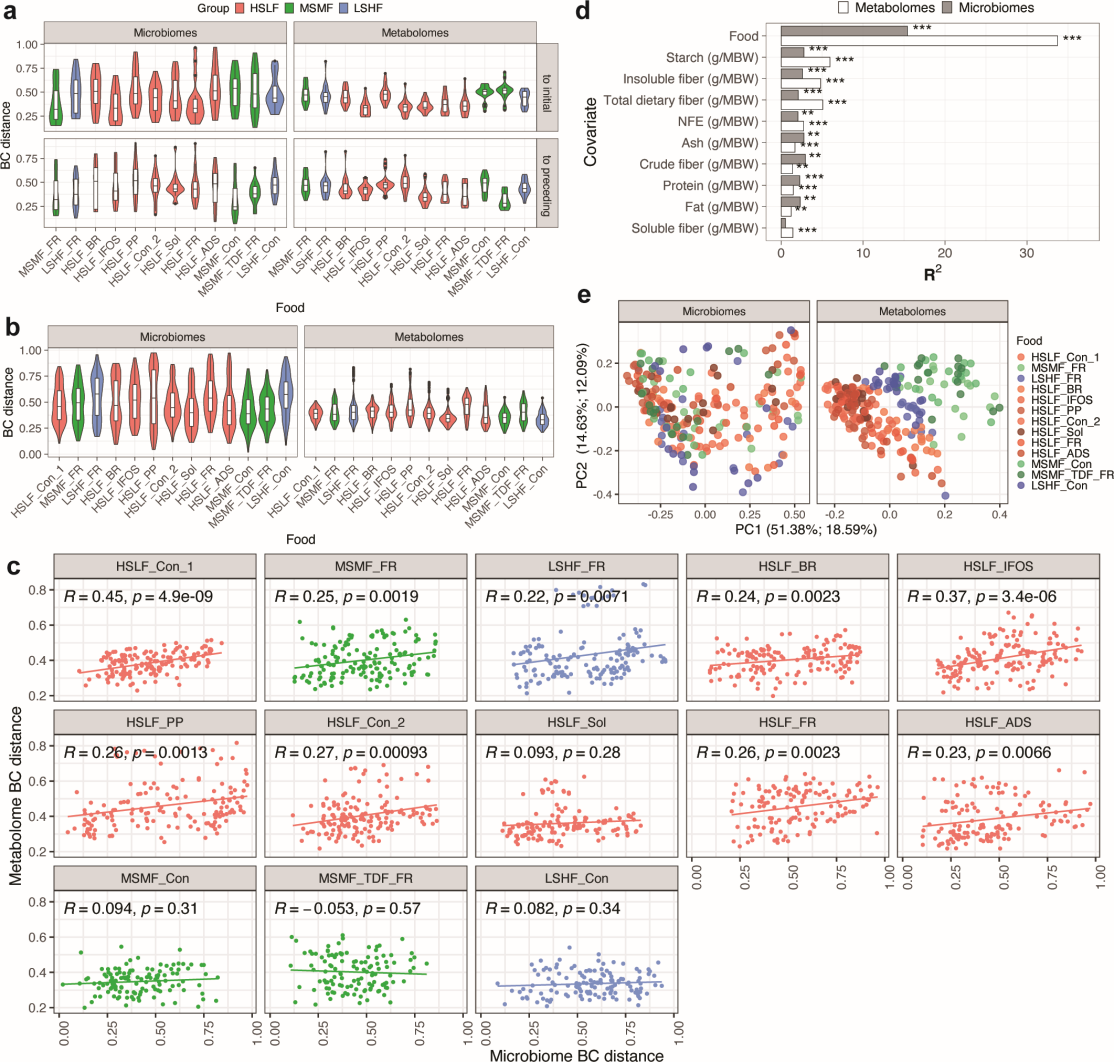
Effect of specific foods on the canine gut microbiome and fecal metabolome. (a) Comparison of the intra-subject BC distances among microbiome taxonomic profiles and metabolomes of the subject population in response to each food in comparison with control food i.e., HSLF_Con_1 (initial) and with the preceding food. *n* = 216 for each box. Metabolomes but not microbiomes corresponding to HSLF foods are more similar to those of HSLF_Con1. Similarly, metabolome in response to the metabolome of the preceding food provided both foods are in the same food group. (b) The microbiomes and metabolomes of all subjects in response to each food were compared (inter-subject, as opposed to the intra-subject data in panel a) to identify foods that induce more similar microbiomes/metabolomes versus those that are more heterogeneous. Overall, metabolomes in response to the same food are more similar than the corresponding microbiomes. *n* = 216 for each box. (c) Relationship between metabolome and microbiome BC distances stratified by food. Pearson correlation coefficients and *P* values are shown. For 8 of the 12 foods, microbiome dissimilarities translate into metabolome dissimilarities. (d) BC univariate PERMANOVA showing components of overall compositional differences in both metabolomes and microbiomes (***P < 0.001 and **P < 0.01). Food, starch, and insoluble fiber explain the most variance in microbiomes and more so, in metabolomes. (e) BC distance-based principal coordinate analysis of microbiomes and metabolomes in response to the test foods shows that metabolomes corresponding to the same food group tend to be more similar than microbiomes. Shades of red, green, and blue represent the three food groups in all panels. ADS, adsorbant; BC, Bray-Curtis; BR, brewers’ rice; Con, control; FR, soluble/insoluble fiber ratio; HSLF, high starch, low fiber; IFOS, inulin, fructooligosaccharides; LSHF, low starch, high fiber; MBW, metabolic body weight; MSMF, medium starch, medium fiber; NFE, nitrogen-free extract; PC, principal coordinate; PERMANOVA, permutational multivariate analysis of variance; PP, plant protein; Sol, highly soluble fiber; TDF, total dietary fiber.

The extent to which the microbiome changed in response to food and whether foods with similar compositions resulted in similar microbiomes and the overall relationships between microbiomes and metabolomes across foods were examined. The microbiome and metabolome of each dog in response to each of the foods were first compared with the initial control (HSLF_Con_1) microbiome and metabolome. The microbiome following consumption of MSMF_FR (MSMF_soluble/insoluble fiber ratio) was most similar to the microbiome following HSLF_Con_1, with a median Bray-Curtis (BC) distance to HSLF_Con_1 of 0.32 (Fig. 2a). Although the composition of MSMF_FR is different from that of HSLF_Con (Fig. 1b), the similarity may be attributable to the sequence of feeding, since MSMF_FR was fed directly after HSLF_Con_1 (Fig. 1a). Among the HSLF foods, consumption of the HSLF_FR and HSLF_IFOS (HSLF_inulin, fructooligosaccharides) resulted in microbiomes most similar to those following consumption of HSLF_Con_1 (both with median BC distance to HSLF_Con_1 of 0.33) (Fig. 2a; Table S6).

In contrast to the microbiome observations, metabolites were more similar when the dogs were fed similar foods (Fig. 2a). For example, the metabolome following consumption of MSMF_FR differed significantly from that following consumption of HSLF_Con_1 (mean BC distance: 0.48; *P* < 0.001). In contrast, consumption of foods that were similar in nutrient composition to HSLF_Con_1, such as HSLF_Con_2 (identical), HSLF_IFOS, HSLF_Sol (HSLF_high soluble fiber), HSLF_ADS (HSLF_adsorbant silicon dioxide), and HSLF_FR, resulted in metabolomes similar to that of HSLF_Con_1 (mean ± SD BC distance: 0.34 ± 0.08, 0.33 ± 0.08, 0.36 ± 0.06, 0.37 ± 0.11, and 0.37 ± 0.11, respectively), indicating that foods with similar nutrient components are reflected by similarity in fecal chemicals (either due to direct pass through or due to similar microbial metabolism). Although the HSLF_BR and HSLF_PP foods were similar in macronutrient composition to HSLF_Con_1, their sources of starch and protein, respectively, were different. Accordingly, their metabolomes were less similar to the HSLF_Con_1 (mean ± SD BC distance: 0.45 ± 0.09 and 0.48 ± 0.09, respectively) compared with the other HSLF metabolomes, indicating that the sources of macronutrients impact colonic chemistry (again, directly, and/or via microbial metabolism), as previously observed (30). Notably, all of the metabolomes following consumption of the MSMF or LSHF foods were even more distinct from the HSLF_Con_1 metabolome compared with HSLF foods (*P* < 0.01 for LSHF and *P* < 0.001 for MSMF; mean BC distance for HSLF, MSMF, and LSHF: 0.39, 0.49, and 0.45, respectively). In summary, consumption of foods with compositions similar to those of HSLF_Con resulted in similar metabolomes but variable microbiomes.

The microbiomes in response to a given food were also compared with the microbiome in response to the food directly preceding it (Table S6). There was no clear trend in the BC distances of microbiomes in response to preceding foods, that is, similar microbiomes were observed following consumption of non-similar foods (HSLF_Con_1 and MSMF_FR; HSLF_ADS and MSMF_Con), and dissimilar microbiomes were observed in response to similar foods (HSLF_FR and HSLF_ADS; Fig. 2a). Overall, adjacency of feeding or similarity in food composition did not have a significant effect on the similarity of microbiomes (Fig. S3). This is again in contrast to the metabolomes, where similarity of food composition was a direct correlate of similarity of colonic metabolites. For example, the metabolomes following consumption of the HSLF foods were more similar to HSLF_Con_1 or to that of another high-starch food directly preceding them than to the low-starch food directly preceding them. Comparison of microbiomes and metabolomes across the foods suggests that individual microbial communities can be robust to dietary perturbation and can provide convergent metabolic potential to yield similar metabolomes from similar foods.

### Microbiome-metabolome relationships in response to dietary change

To better understand this functional robustness of the gut microbiome, microbiomes and metabolomes that arose in response to the same food were compared by examining pairwise BC distances (Fig. 2b; Table S6). Overall, as implied by the analyses above, the distribution of pairwise BC distances for the microbiomes were wider than those for the metabolomes, indicating that very different microbial communities can correspond with similar sets of metabolites, reiterating the observations from intra-subject comparisons of foods. Although of lesser effect than changes in metabolomes, consumption of MSMF_Con (mean ± SD BC distance: 0.42 ± 0.17) gave rise to most similar microbial communities among the dogs, whereas LSHF_Con (mean ± SD BC distance: 0.57 ± 0.19) caused microbiomes to be most dissimilar. For the metabolomes, consumption of LSHF_Con (mean ± SD BC distance: 0.37 ± 0.07) resulted in harmonization, while consumption of HSLF_FR (0.46 ± 0.1) and HSLF_PP (0.46 ± 0.13) resulted in the greatest diversification of the metabolomes.

Next, the degree to which differences among microbiomes resulted in differences among metabolomes was tested. Overall, taking all foods into consideration, a weak but significant positive correlation was seen between pairwise microbiome and metabolome BC distances (Spearman *r* = 0.29, *P* < 0.0001), meaning that, to a degree, more similar microbiomes corresponded with more similar metabolomes (Fig. S4). Alpha diversity variation was even less consistent among foods, and alpha diversity either remained the same or decreased (Fig. S5), as previously observed in human dietary interventions (37, 38). Correlations were stratified by food to evaluate the effect of the microbiome on the metabolome, which also highlights food components that are broadly versus specifically metabolizable (i.e., which foods have components that can be metabolized by most members versus only some members of the microbiome; Fig. 2c). Notably, for foods such as MSMF_Con, MSMF_TDF_FR, LSHF_Con, and HSLF_Sol, the differences in microbiomes did not translate into differences in metabolomes, indicating metabolism by the same pathways in different microbes or by microbiome-independent routes. On the other hand, for 8 out of 12 foods, more differentiated microbiomes correlated with more differentiated metabolomes, indicating the presence of pathways specific to only some community members. These functional differences affect the abundances of common diet-linked, microbe-derived compounds such as the products of carbohydrate and protein metabolism (based on the abundance of respective metabolizing species) or the presence and abundances of more unique metabolites that are produced from unique dietary substrates by a very small fraction of community members encoding specialized functions.

Despite the relative robustness of individual microbiome composition across changing foods, in this controlled context, food did correspond with the greatest component of variance in metabolomes (33.7%) and second highest for microbiomes (15.4%) as measured by permutational multivariate analysis of variance (PERMANOVA) (Fig. 2d). Variance explained by inter-individual heterogeneity was higher than diet for gut microbiomes (28.6% versus 15.4%) but lower than diet for fecal metabolomes (18.5% versus 33.7%), as expected. All other macronutrient intakes explained smaller but notable variances, with starch intake being highest among these (*R*^2^ = 2.8%, *P* < 0.001). For starch, insoluble fiber, and total dietary fiber, the variance explained for metabolomes was larger than that for microbiomes (6% vs 2.8%, 4.8% vs 2.6%, and 5% vs 2%, respectively). Conversely, crude fiber, protein, fat, and ash explained more of the variance for microbiomes than for metabolomes (albeit still with small percentages, maximum *R*^2^ = 2.9%). This observation lends support to a hypothesis in which rapidly accessible nutrients have growth-based enrichment effects on microbes with different fixed protein- and fat-metabolizing capabilities, whereas transcriptional and other regulatory mechanisms are more important in polysaccharide (starch, fiber)-metabolizing capabilities.

Although no single aspect of the foods had a consistently strong effect on microbiome composition across the whole population, starch and insoluble fiber contents were the primary determinant of metabolome similarity (Fig. 2e; Fig. S6). Microbiome structure was not strongly associated with low- versus high-starch diets (Fig. 2e; Fig. S7), but microbiomes resulting from consumption of foods containing similar protein and fat content were more similar, although to a very moderate degree (PERMANOVA *R*^2^ 2.28% and 2.32%, respectively). Collectively, these results demonstrate the complexity of gut microbial regulatory networks providing robustness in the face of changing diets and thus that multiorganism metabolic networks can convert different substrates from food into similar chemical outputs, at least at a gross energy harvest level.

### Microbial and metabolite features are associated with dietary macronutrients

**Fig 3:**
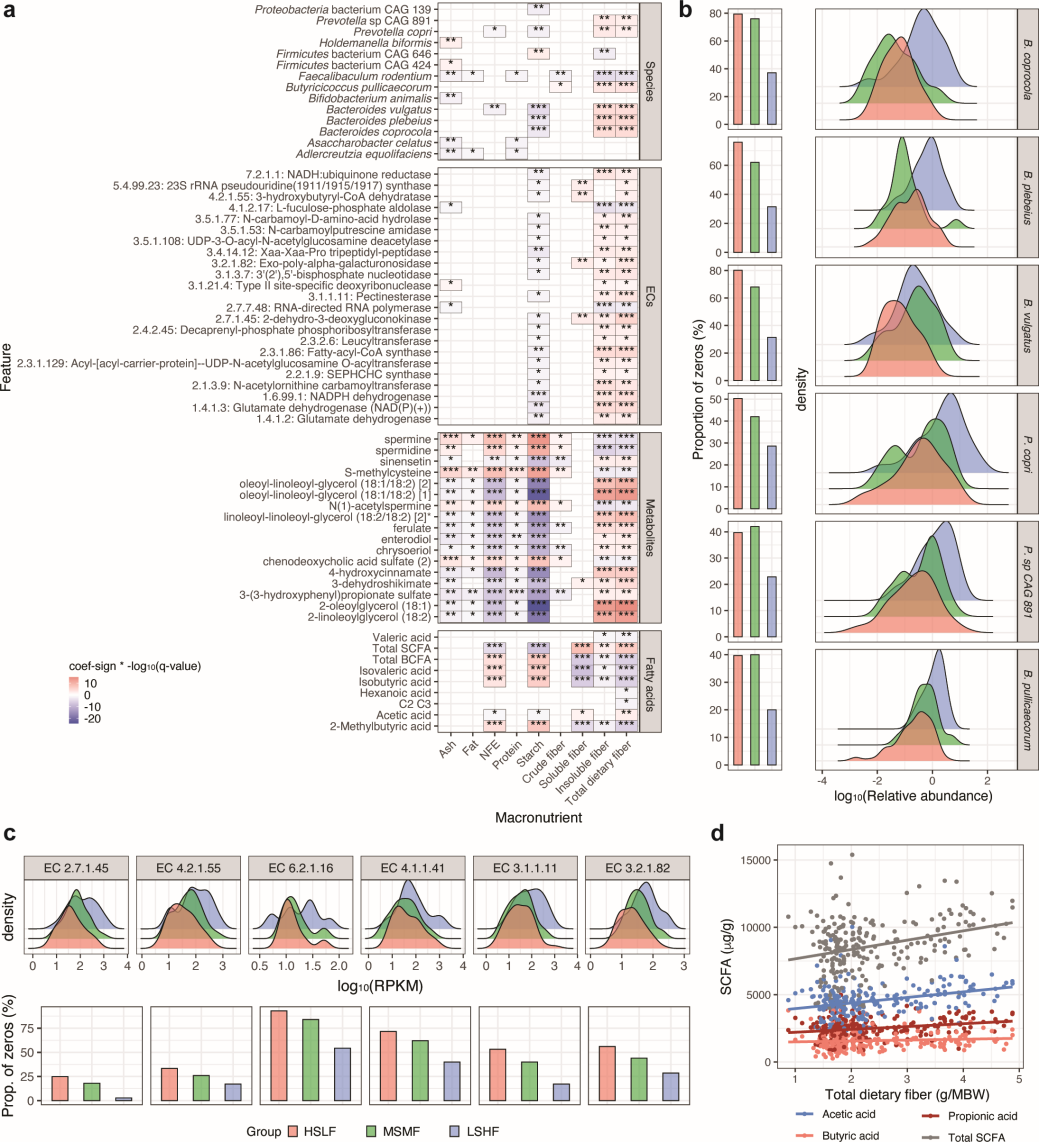
Associations between canine gut microbial and metabolic features and dietary macronutrients. (a) Significant associations between gut microbial and metabolic features and dietary macronutrients determined using univariate linear models, with macronutrient intake as the fixed effect and subject as the random effect. A filtered list is shown for species (*q* value < 0.05, coefficient > 50th percentile), enzymes (*q* value < 0.05, coefficient > 50th percentile that are associated with ≥3 macronutrients), fatty acids (*q* value < 0.05), and metabolites (*q* value < 0.05, coefficient > 50th percentile that are associated with ≥7 macronutrients). Complete results are in Table S7. (***q < 0.001, **q < 0.01, and *q < 0.05). (b) Log_10_ (relative abundance) of fiber-responsive bacterial species of *Bacteroides*, *Prevotella*, and *Butyricicoccus* following consumption of the HSLF, MSMF, and LSHF foods. (c) Log_10_ (RPKM) of enzymes involved in SCFA synthesis (butyrate: EC 2.7.1.45, EC 4.2.1.55, and EC 6.2.1.16; propionate: EC 4.1.1.41) and pectin degradation (EC 3.1.1.11 and EC 3.2.1.82) following consumption of the HSLF, MSMF, and LSHF foods. Enzyme names as in panel a. (d) Total SCFA and individual SCFAs are correlated with total dietary fiber intake (g/MBW). Total SCFA: R = 0.31, *P* < 0.001; acetic acid: R = 0.33, *P* < 0.001; propionic acid: R = 0.27, *P* < 0.001; butyric acid: R = 0.091, *P* = 0.17. CAG, co-abundant gene; EC, Enzyme Commission; HSLF, high starch, low fiber; LSHF, low starch, high fiber; MBW, metabolic body weight; MSMF, medium starch, medium fiber; RPKM, reads per kilobase of exon per million reads mapped; SCFA, short-chain fatty acid.

When examining associations between individual microbial features (species and enzymes), metabolic features (fecal metabolites and SCFAs), and dietary macronutrients at a greater level of specificity, several potential interactions were identified. Overall, associations between individual metabolites and macronutrients were again found to be both stronger and more numerous than those between individual microbial features and macronutrients (Fig. 3a; Table S7). Twenty-five and 20 species, which spanned multiple phyla, were associated (*q* < 0.25) with insoluble fiber and starch, respectively. These included three *Bacteroides* (now genus *Phocaeicola*) spp. (*Bacteroides coprocola*, *Bacteroides plebeius*, and *Bacteroides vulgatus*), two *Prevotella* spp. (Prevotella sp. CAG 891 and *Prevotella copri* now renamed to *Segatella copri*), *Butyricicoccus pullicaecorum*, *Lachnospira pectinoschiza*, and a Proteobacteria co-abundance gene group (CAG) (Fig. 3a; Table S7). Compared with HSLF, these species were more abundant following consumption of the LSHF foods and, to a lesser extent, MSMF, both of which contained higher percentages of insoluble fiber relative to HSLF (Fig. 3b; Table S7). In contrast, Firmicutes were broadly negatively associated with insoluble fiber, including *Faecalibacterium rodentium*, *Dubosiella newyorkensis*, and a Firmicutes CAG.

A large number of individual enzymes (*n* = 189; almost 10% of the total enzymes detected from metagenomic sequences) identified by Enzyme Commission (EC) numbers were also significantly (*q* < 0.05) associated with insoluble fiber (Table S7). In contrast, only 17 were associated with soluble fiber. Interestingly, insoluble fiber-associated ECs included enzymes involved in the metabolism of the soluble fibers pectin (EC 3.1.1.11, EC 3.2.1.82), glucan (EC 3.2.1.58), and pullulan (EC 3.2.1.41) (Fig. 3a and c; Table S7).

In addition, an enrichment of enzymes involved in the biosynthesis of the butyrate (EC 2.7.1.45, EC 4.2.1.55, and EC 6.2.1.16) and propionate (EC 4.1.1.41) was observed in overall high-fiber foods (Fig. 3a and c). When fecal SCFA levels were measured directly, total SCFA was positively associated with both soluble and insoluble fiber. Of individual SCFAs, only acetic acid was significantly positively associated with soluble fiber (*q* < 0.05) and to a lesser extent with insoluble fiber (*q* = 0.05) (Fig. 3a; Table S7). However, a substantially non-uniform increase in individual SCFAs was observed in response to increases in total dietary fiber consumption (Fig. 3d). Conversely, both total branched-chain fatty acids (BCFAs) and individual BCFAs were significantly associated with starch intake (Fig. 3a). Other metabolites of protein metabolism, such as the polyamines putrescine and spermine, were also positively associated with starch content (Fig. 3a; Table S7).

Several acylglycerols were significantly enriched in metabolomes corresponding to consumption of high total dietary fiber (Fig. 3a). Correspondingly, fatty acyl CoA synthase (EC 2.3.1.86) and glycerol were also significantly positively associated with total dietary fiber (Table S7). Insoluble fiber degradation products, including sugars such as arabinose, xylulose, xylose, and fucose, and polyphenols, such as diosmetin, enterodiol, genistein, hesperidin, hesperetin, limonin, secoisolariciresinol diglucoside, secoisolariciresinol, and naringenin, were significantly positively associated with total dietary fiber (Table S7).

### Fiber-microbe associations influence the colonic chemistry of dietary intake

**Fig 4:**
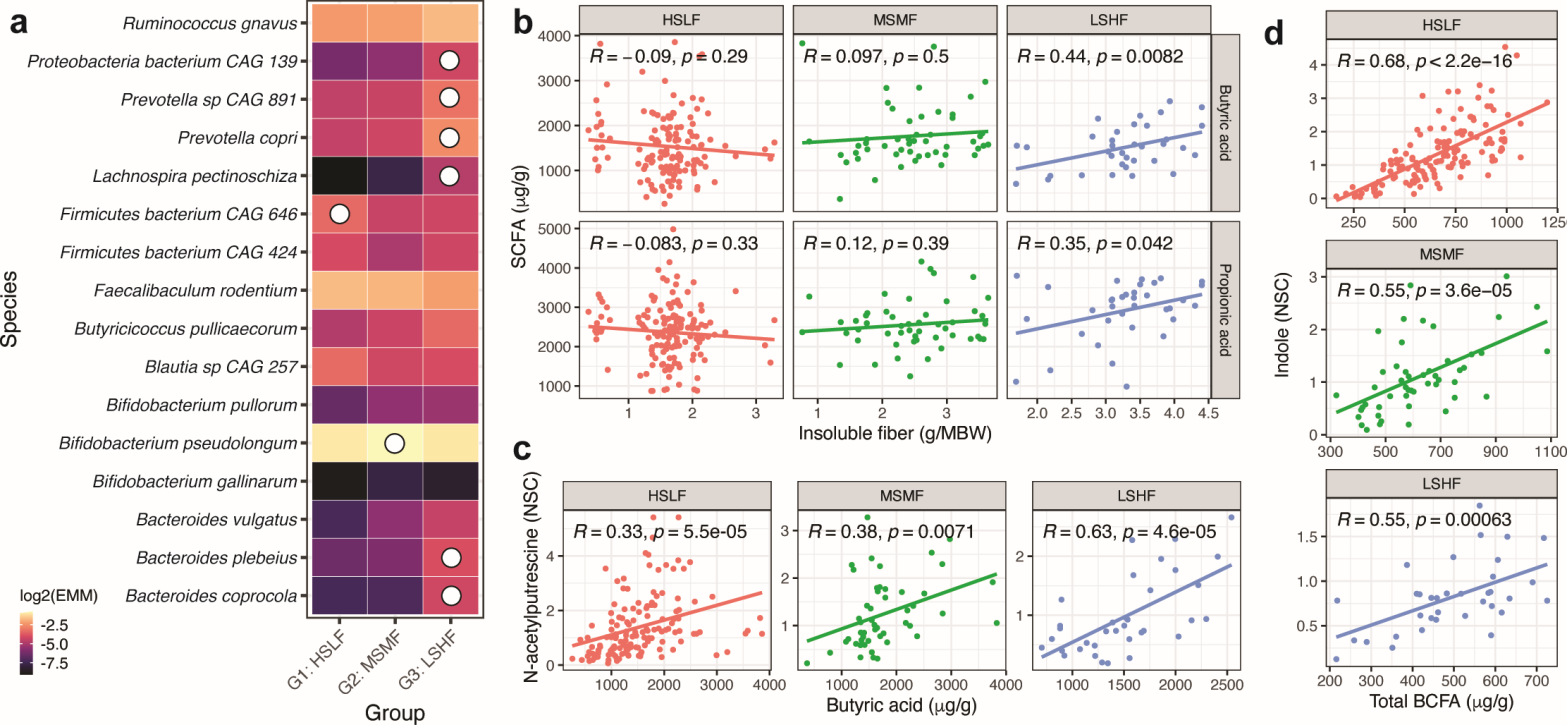
Heterogeneity in microbial responses to dietary fiber. (a) Microbial species (*n* = 16) differentially abundant among food groups were identified by mixed-effect linear models and comparison of EMMs. White circles indicate species (*n* = 8) enriched in only one group. (b) Association between the SCFAs butyric acid/propionic acid and insoluble fiber, stratified by food groups. SCFAs are significantly associated with insoluble fiber intake only in LSHF foods. (c) Association between beneficial microbial metabolites N-acetylputrescine and butyric acid across food groups. (d) Association between products of protein degradation (indole and total BCFA) across food groups. CAG, co-abundant gene; HSLF, high starch, low fiber; LSHF, low starch, high fiber; MSMF, medium starch, medium fiber; NSC, normalized spectral counts; SCFA, short-chain fatty acid.

The chemical fates, defined as the metabolism to health-relevant short chain fatty acids, of different types of fiber sources among the study foods as they interacted with the gut microbiome were investigated. First, we analyzed differences in the abundances of taxa across the three test food groups using species-wise linear mixed-effect models and estimated marginal means (EMMs) of a species in the food groups (see Materials and Methods). Sixteen species were differentially abundant (adjusted *P* value < 0.1) across the food treatment groups (Fig. 4a). Most of the differentially abundant species (*n* = 12) occurred between the microbiomes resulting from consumption of LSHF and HSLF (Table S8). For other comparisons, the numbers of significantly differentially abundant species were seven (MSMF and HSLF) and nine (LSHF and MSMF). Of the 16 total differentially abundant species, 8 were enriched specifically in one food group, primarily 6 species in LSHF (i.e., insoluble fiber responsive), similar to those indicated via feature-wise macronutrient-association tests above (e.g., *Bacteroides* species, *Prevotella* species, *Butyricicoccus pullicaecorum*, and *Lachnospira pectinoschiza*; Fig. 3a).

Since these microbial responses to LSHF and other food groups occurred only within a subset of individual subjects (as further investigated below), the effect of microbial responses (when present) on subsequent metabolite production was tested. Interestingly, the association between butyric and propionic acids and insoluble fiber was strongest in LSHF of all the test foods, in which enrichment of *Butyricicoccus pullicaecorum* and Bacteroidetes was observed (Fig. 4b). The strength of association between beneficial microbial metabolites such as N-acetylputrescine and butyric acid (Fig. 4c) and products of microbial protein degradation such as indole and BCFAs (Fig. 4d) varied across food groups.

### Response to dietary fiber is subject specific

Inasmuch as no two canine gut microbiomes are ever identical, the extent of individualization is much lower in facility-housed dogs in this study (mean BC distance of baseline gut microbiomes of 18 dogs in this study: 0.48 ± 0.16) compared with free-living humans (mean BC distance of 26 healthy human gut microbiomes in the HMP2 (35): 0.74 ± 0.16] (Fig. S2). Even in this relatively homogeneous animal population, both chemical and (especially) microbial shifts of subjects in response to the same food were often quite different (Fig. 2b). Thus, the degree of individualization in microbial and metabolic responses to the test foods was examined across subjects by stratifying significant (*q* < 0.05) associations of species and the top 25 metabolites with dietary macronutrients (insoluble fiber, soluble fiber, total dietary fiber, and starch) by subject. That is, only measurements from a single subject were considered to determine the extent to which each subject’s ecology responded to dietary changes.

**Fig 5:**
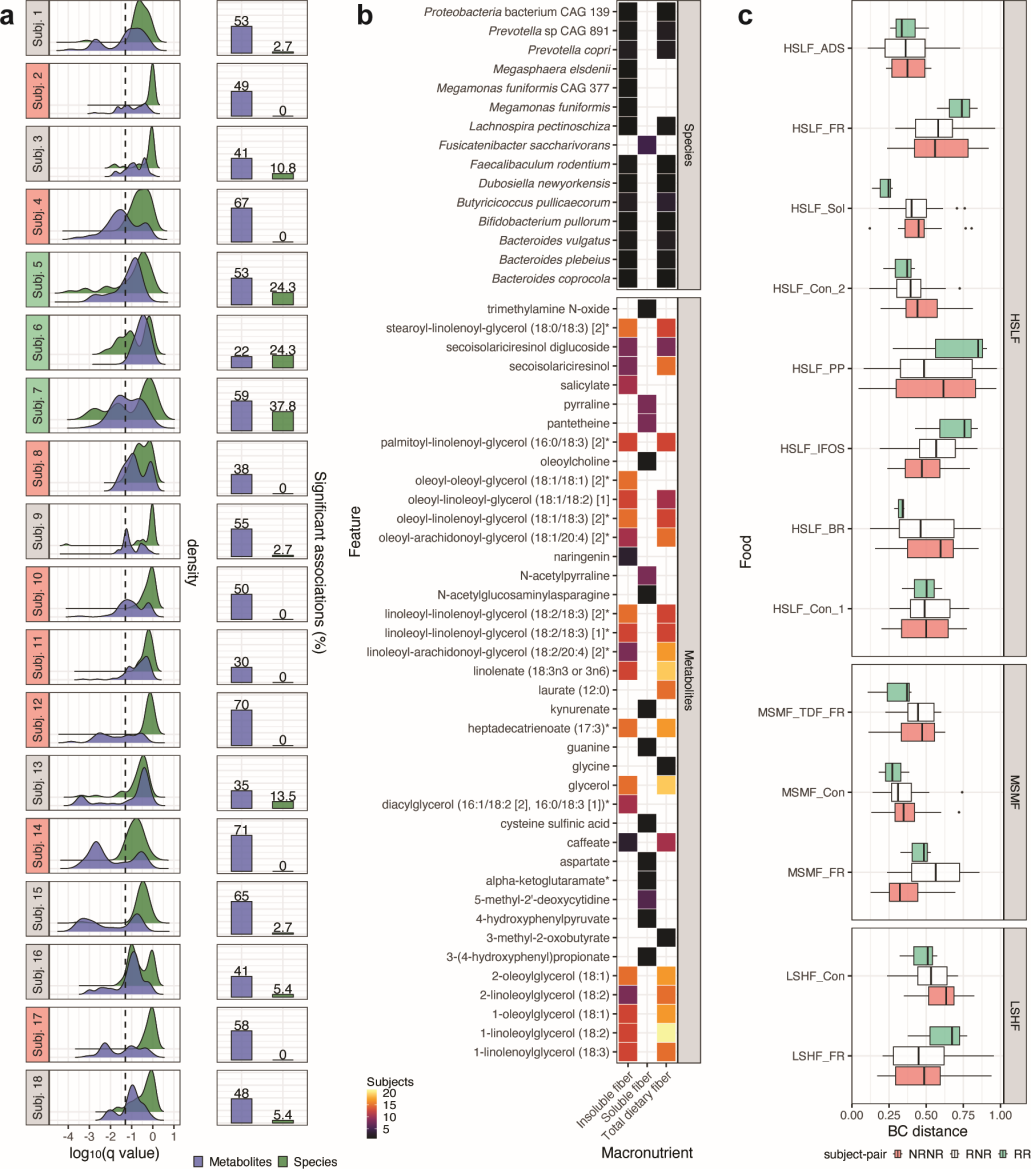
Metabolome and especially microbiome responses to food are highly individualized even in a relatively homogeneous companion animal population. (a, left) Subject-stratified distribution of *q* values for associations of insoluble fiber, soluble fiber, total dietary fiber, and starch (jointly) with microbial (*n* = 37) and metabolic features (*n* = 100; top 25 significantly associated with each macronutrient) previously identified to be significantly associated at the population level (see Fig. 3a). For subject labels, green denotes responders, pink non-responders, and gray neither. (Right) Percentage of species and metabolites significantly associated with fiber or species in each dog. (b) Consistency of significant associations between species/metabolites and fiber across animals. (c) Distribution of pairwise BC distances within and between response categories. ADS, adsorbant; BC, Bray-Curtis; BR, brewers’ rice; CAG, co-abundant gene; Con, control; FR, soluble/insoluble fiber ratio; HSLF, high starch, low fiber; IFOS, inulin, fructooligosaccharides; LSHF, low starch, high fiber; MSMF, medium starch, medium fiber; NRNR, comparison between non-responders; PP, plant protein; RNR, comparison between a responder and non-responder; RR, comparison between responders; Sol, highly soluble fiber; TDF, total dietary fiber.

Overall, the percentage of associations that retained significance (*q* < 0.05) at the subject level was higher for metabolites than for species (Fig. 5a). Per subject, the percentage of significant associations ranged between 22% and 71% for metabolites and 0% and 38% for species (Table S9). Subjects 1, 4, 9, 10, 12, 14, 15, and 17 with poor microbiome response (>95% non-significant *q* values) showed high metabolite response (≥50% significant *q* values; Fig. 5a). In contrast, the subjects with the three highest microbiome responses (subjects 5, 6, and 7) showed 22%–59% significant metabolite responses. Four of the top five metabolite response subjects had poor microbiome responses. Notably, these differences were observed despite relatively modest baseline differences in microbiome profiles (Fig. 1) or biometrics, as well as well-controlled animal environment and housing. Significant associations between taxa and insoluble fiber were more consistently observed than associations with soluble fiber (Fig. 5b). Of the positive associations between metabolites and macronutrients, associations between insoluble fiber were also more consistent than those with soluble fiber (Fig. 5b).

Based on the total number of significant associations observed for each subject between species and insoluble/soluble/total dietary fiber (*n* = 30), three subjects (subjects 5, 6, and 7) were classified as responders and eight as non-responders (Fig. 5a). Specifically, all 30 possible food-microbiome associations were non-significant in non-responders, whereas responders had ≥25% significant associations. Responders did not show apparent bias for age (13.6, 9.2, and 5.1 years of age) or sex (one male, two females). To ascertain if responder microbiomes were more similar to each other, for each food, pairwise microbiome BC distances were compared among responders, non-responders, and responders and non-responders. There was no trend in the distribution of BC distances across test foods or food groups (Fig. 5c). Moreover, abundances of fiber-reactive Bacteroidetes (Fig. 3a and 4a) were similar in both responders and non-responders in response to control (HSLF_Con) food (Fig. S8). Lastly, food intakes were examined to determine whether microbiome response was intake dependent, i.e., whether consumption of more insoluble fiber led to more significant associations. There were no observed differences in the intakes of responders and non-responders (Fig. S9).

## DISCUSSION

In this study, we examined paired fecal multiomic data associated with consumption of various fibers in a canine population to understand how specific microbiota-macronutrient associations underlie metabolic benefits from diet. This experimental setting represents a scenario of intermediate complexity between free-living human populations and fully controlled laboratory animals (19). Here, the food being consumed corresponded with the greatest component of variance in microbiome composition in this study (~16%), corroborating this expectation, as this is greater than that observed in most human populations (<1%–10%) (39–41), but less than in laboratory mouse feeding experiments (~35%) (6). This permitted quantification of personalized microbial and metabolic responses to fiber intake (42).

Prior studies of human microbiome associations with soluble fiber broadly concur in diverse enrichment of Firmicutes, most commonly *Faecalibacterium prausnitzii* (9, 43, 44) and *Roseburia* (9, 43, 45). On the other hand, insoluble fiber supplementation is generally supportive of Bacteroidetes and Actinobacteria enrichment (12, 46–48). Beyond these phylum-level expectations, however, this study’s finer resolution allowed us to observe enrichment of both Bacteroidetes (*Bacteroides* and *Prevotella* spp.) and Firmicutes (*Butyricicoccus* and *Lachnospira* spp.) in response to insoluble fiber intake among different subjects (Fig. 3a). *Fusicatenibacter saccharivorans* was the only species that was consistently positively associated with soluble fiber in this population.

Diversity of dietary and health history is also likely to have a substantial effect on subject-specific microbial responses to food (49), since the range of differences experienced by most, but not all (5), human populations over a lifespan is much greater than that experienced by canines, allowing the evolution of microbial ecological resilience (50). Accordingly, metabolome responses to food were quicker, stronger, more numerous, and much more consistent among subjects than were those of microbiota. At the population level, associations between fiber intake and metabolic features including SCFAs and several acylglycerols were observed. Total SCFA, for example, was positively associated with both soluble and insoluble fiber, with overall relative proportions of individual SCFAs as previously reported (i.e., acetate > propionate > butyrate) (51).

Notably, increased dietary fiber did not always correspond with increased individual SCFA production (Fig. 4b). This occurred only when (i) a species capable of producing a particular SCFA in excess was already present and (ii) its enriched growth was supported by a particular fiber source. *Butyricicoccus pullicaecorum* can predominantly produce butyrate (52), *Lachnospira pectinoschiza* is a predicted butyrate producer (53), and many Prevotella and Bacteroides are likely propionate producers (54). In this context, *Butyricicoccus pullicaecorum* was enriched in MSMF and LSHF foods whereas *Prevotella*, *Bacteroides*, and *Lachnospira pectinoschiza* were enriched only in LSHF, likely due to the abundance of pectin, arabinan, and other plant fibers and metabolites that are contributed by peas, beets, and tomatoes that are unique to these foods (Table S2) (55–57). A previous study has reported that several *Bacteroides* species including *B. vulgatus* (enriched in high-fiber foods; Fig. 4) contain polysaccharide utilization loci (PUL) that encode enzymes to utilize arabinan in pea fiber (57). Similarly, human gut isolates of *Megasphaera elsdenii* (significantly positively associated with insoluble fiber in our study; Table S7) were found to encode carbohydrate-degrading enzymes (CAZymes) (58). Correspondingly, an interesting potential explanation for the diversity of responses to insoluble fiber could be the highly varied carriage of genes encoding CAZymes among members of the aforementioned phyla, which drive specialization in the utilization of complex plant-derived polysaccharides (47, 59–61). Not only are CAZymes highly substrate specific but their carriage among these clades is diverse from the phylum to strain levels, leading to differing microbial metabolic strategies that are difficult to characterize from either taxonomy or enzyme sequences alone. Put together, this suggests a differential benefit cascade in response to different fiber sources, macronutrient levels, and microbial carriage. That is, the chemical (and presumably phenotypic) consequences of a food are a function both of its composition and of how differing pre-existing or responsive microbes metabolize it, analogous to context-specific microbe-fiber interactions observed in healthy and diseased human populations (45, 49).

Previous studies have linked specific PULs with particular fibers (57), which would suggest these fibers to be predominantly bioactive only in individuals whose microbiomes carry these PULs. Similarly, other studies have identified factors including *Helicobacter* species, microbial hydrogen cyanide synthases, and serum proteins that affect benefit from fiber consumption; specifically, the levels of low-density lipid (LDL) (62). Thus, future efforts may combine information gathered from metagenomes (fiber-metabolizing species, enzymes, and species interfering with fiber metabolism) and other biomarkers such as serum proteins, triglycerides, and protein:fiber ratio in diet to inform personalized dietary fiber recommendations in both companion animals and humans. While this study’s population size and longitudinality are substantial in comparison with previous canine dietary supplementations, the scope of the population is likely still smaller than will be necessary to understand the individualized microbial chemistry and regulation driving metabolomic responses. Previous studies have implicated associations between breed size, intestinal length, transit time, and gut microbiome, with implications for digestion efficiency and realization of microbiome-derived benefits from dietary components (63). Our study was designed to screen for response to fiber predominantly in medium-sized canines; future investigations will include breed size as a factor by design as well as more subjects.

Other limitations include the lack of a randomized diet order, no washout period between the different test foods, and short (7-day) feeding periods per food, precluding the ability to completely differentiate adjacency effects from those of some individual diets. Future investigations will include a study design that accounts for carry-over effect (e.g., Latin square), longer study duration per food, and a wash-out period. The degree to which different test foods differed only slightly in composition can be considered either a strength or a weakness, since it helps in pinpointing the likely nutritional origins of metabolomic responses to those foods but, conversely, does not enrich for strongly differentiated responses. The observed phyla in this study broadly agreed with the canine gut microbiome (64); however, the increased levels of Actinobacteria compared with those of previous studies (33, 34) are probably due to a facility effect (e.g., environment or dietary history), as is common in mouse and other animal housing facilities (65, 66). Finally, other uncontrolled factors such as housing, physical activity, host genetics, and diet history may impact the variability of fiber response in a population.

Therefore, for population-wide dietary fiber recommendations, variability in host dietary histories (67), physiologies (62), and gut microbiome compositions (45, 68) are important considerations. In other words, for a more robust understanding of response, we need more data sets that take baseline microbiome functional diversity and metabolic capabilities as well as relevant host biomarkers into account (69, 70). No fiber is expected to be equally beneficial for all individuals, future studies can explore fibers that will be beneficial for populations corresponding to specific phenotypes or metabolic states, i.e., fibers that are beneficial for obese versus lean individuals or fibers that are suitable for high-fiber consumers. Even then, variability in response to fiber is expected. Finally, the response to a particular dietary fiber intervention might best be defined by uniformly common metrics such as abundance of health-relevant microbial products of fiber metabolism, as well as metabolic, immune, and physiological improvements in addition to community expansion of fiber-responsive taxa.

Along with the overlap in fiber-responsive metabolites and species between the two hosts, which makes the findings in canine populations translatable to humans, the individualized microbial response to food like in humans (42) was perhaps the most intriguing outcome of this study, even after controlling population features, dietary composition, intake, and environmental factors (e.g., housing and outdoor exposure). These data also suggest two potential determinants of personalized food-microbiome-metabolome interactions. There could be subtle, rather than overall, differences in baseline microbiome composition (specifically, fiber degraders), such that low-abundance community members influence key functions. These would not be detectable in the relatively small population studied here. Alternatively, microbially influenced, but not compositionally encoded, factors such as host immune memory and transcriptional regulatory state may explain these personalized interactions (not analyzed in this study). Differentiating between these will be crucial for the success of human personalized nutrition, which has been the focus of both population epidemiology and commercialization. Larger and more targeted studies will allow explanatory or prognostic biomarkers to be identified, enabling direct prediction of effective, personalized dietary interventions for beneficial metabolic outcomes.

## MATERIALS AND METHODS

### Study foods and compositions

The 12 foods described in this study, including the control food, were manufactured at the Hill’s experimental food laboratory (Pet Nutrition Center, Topeka, KS, USA). Compositions of the foods were all in dry form and formulated to meet the maintenance nutrition requirements recommended by the Association of American Feed Control Officials (AAFCO) (71). The control food (HSLF_Con) was formulated to be similar to commercially available foods recommended by veterinarians for the management of canine gastrointestinal and dermal diseases. A characteristic of these foods is their inclusion of pre-hydrolyzed protein as the source of amino acids and inclusion of purified starch rather than intact grains as the source of carbohydrate. Predigested protein and purified starch are included to improve digestibility of these foods, and indigestible fiber is kept low. Hydrolyzed protein has shown efficacy in the management of food allergens (72). Foods contained cornstarch as the primary source of digestible carbohydrate (starch) and chicken liver hydrolysate as the primary protein source, which provide less bypass digesta for microbiome metabolism relative to typical canine foods. In order to maintain appropriate stool firmness, the control food also contained cellulose as a source of fiber that is relatively inert in relation to microbiome fermentability.

A previous study found differences in the impact of a blend of fiber on the canine gut microbiome when included in diets that did or did not contain hydrolyzed protein (14), but that study only examined a single blend of fiber. The current study used a hydrolyzed protein-containing, high starch, low fiber control food, which served as a base to include additional fibers (other foods in the study) and examine how these fibers differentially modulate the canine gut microbiome when included in a therapeutic food formulation commonly employed to mitigate disease. In HSLF_BR, brewers’ rice replaced cornstarch to increase type 2 resistant starch (RS2) due to the more complex structure of the native carbohydrate source. In HSLF_PP, soy protein hydrolysate replaced chicken liver hydrolysate. The HSLF_Sol food contained 4% by weight of digestion-resistant maize dextrin (RS4), a prebiotic, soluble, non-viscous fiber produced by selective hydrolysis, and repolymerization of starches from wheat (73). The remaining foods used in this study were formulated identically to HSLF_Con except that different fiber sources at various levels were substituted for cornstarch and cellulose (Table S2). The fibers were chosen based on their solubility and predisposition toward fermentation by colonic microbes (4).

In addition to being the preferred source of starch in veterinary foods for canine gastrointestinal and dermal diseases, cornstarch as the operative nutritional carbohydrate source provided an opportunity to reduce levels of residual-resistant starch in the foods that might be variably present after food processing. Minimization of residual-resistant starch (except for the intentional case of HSLF_BR) was done with the intent to make more readily apparent the impact of the fiber interventions (74), as they would be less encumbered by confounding effects of resistant starch. Cornstarch is highly refined and thus devoid of the sort of resistant starch that is protected from digestion by being tightly ensconced within the digestively impenetrable confines of seed coatings (RS1) (75). When purified starches such as cornstarch are extensively processed via extrusion, they retain little native starch granule structure (RS2). Extruded pet foods are dried extensively before cooling and thus undergo relatively little retrogradation of starch during cooling and storage; thus, they would not be expected to contain appreciable amounts of RS3.

Chicken liver hydrolysate was utilized as the primary protein source because hydrolytic pre-digestion of the protein is expected to improve digestibility in the upper gastrointestinal tract and reduce the amount of digestion-resistant protein that enters into the colon (the milieu of the microbiome) (76). Even when minimizing the quantitative amount to which a protein ingredient delivers digestion-resistant protein to the colon, the qualitative amino acid makeup of bypass protein may influence the composition of microbial putrefactive products. The replacement of chicken liver hydrolysate with soy protein hydrolysate in HSLF_PP controlled for the qualitative amino acid composition.

### Animals and experimental design

The study protocol was approved by the Hill’s Institutional Animal Care and Use Committee (CP14.0.0.0-S-C-MULTI-DIG-MULTI-14-MULTI) and conducted in compliance with the guide for the care and use of laboratory animals from the U.S. National Research Council (77).

Dogs in the study had no evidence of chronic systemic disease from physical examination, complete blood count, serum biochemical analyses, urinalysis, or fecal examination for parasites. Exclusion criteria were recorded instances of gastrointestinal upset (vomiting, diarrhea), known gastrointestinal abnormalities, compromised renal function, abnormally low appetite, or history of food allergies and antibiotic treatment. Dogs remained in their preferred housing arrangement during the trial as previously determined by the colony veterinarian’s assessment of temperament and social interactions. The dogs were housed in pairs overnight for sleeping arrangements, i.e., each dog in this study had a kennel companion that did or did not belong to this study. The study implementation permitted normal socialization and enrichment activities for the dogs. This included daily group exercise in outdoor grassy runs including exposure to seasonal factors. All dogs had the opportunity to exercise and interact together in large groups (~20 dogs). All dogs were immunized against canine distemper, adenovirus, parvovirus, *Bordetella*, and rabies; were monitored for parasites; and received routine heartworm preventative. Prior to initiation of this study, all dogs had consumed the same food (maintenance food of Hill’s Science Diet Adult) with composition distinct from any included here.

The feeding study was performed as a non-randomized sequential design in which all dogs were fed a given food for a period of 7 days [since previous studies detected changes in the gut microbiome within a week of food intervention in both dogs and cats (78, 79)] and moved to the next assigned food. There was no washout period between consecutive foods. Foods were fed in sequence starting with the control (HSLF_Con) food (Fig. 1a). Each dog was fed an amount of each food based on their daily caloric requirements as calculated based on their body weight (post hoc measured intakes reported in Table S2). Dogs were each fed individually once daily in the morning (before 10:00 hours) in individual feeding stations. Food intake amount (g/day) for each dog was collected through electronic feeders using a weight scale where each individually identified dog (through a radio-frequency identification chip reader) was given access for 30 minutes to a controlled amount of food calculated to maintain weight. Water was available *ad libitum*. Dogs were weighed weekly.

### Sample collection

Fecal samples were collected as a whole stool from each dog at the end of each feeding period (day 7) within 30 minutes of defecation and transferred into doubled plastic bags, homogenized thoroughly by hand without any visible clumps, aliquoted into 2-mL cryovials, and stored at −70°C prior to further analyses.

### Metabolomic profiling

Analysis of fecal metabolites and short-chain fatty acids was performed by a commercial laboratory (Metabolon Inc., Morrisville, NC, USA). Briefly, fecal metabolite samples were prepared by a proprietary methanol-based solvent extraction method and measured in a randomized order (80) via four different methods using ultraperformance liquid chromatography (ACQUITY UPLC; Waters, Milford, MA, USA) and high-resolution/accurate mass spectrometer interfaced with a heated electrospray ionization (HESI-II, Q-Exactive, Thermo Scientific, Waltham, MA, USA). Raw features were detected and processed via Metabolon’s standards for metabolite identification and quantified by peak area integration. Metabolites not detected in a given sample were imputed with the observed minimum for detection of that metabolite. Fecal SCFAs were separated from fecal matter by liquid-liquid extraction under basic conditions with inclusion of an internal standard, and the resulting extracts were resolved by capillary gas chromatography with flame ionization detection as previously described (14).

In total, 818 metabolites were detected and quantified in the stool metabolomes. Chemical taxonomy of these metabolites was obtained from the Human Metabolome Database version 5 (81) and defined by ClassyFire (82): 511 metabolites were assigned a chemical subclass. The COV of each metabolite was calculated as the ratio of the standard deviation and mean of its abundances in the baseline (i.e., in response to control food HSLF_Con) metabolomes of the 18 dogs. Furthermore, we calculated the median COV of each of the 95 subclasses (i.e., the median value of the metabolites assigned that subclass). To compare the metabolomes of healthy dogs with those of healthy humans, we used the healthy subset of stool metabolomes (*n* = 34; cross-sectional) from the previously published PRISM data set (36). Chemical taxonomy and COV analyses were performed for the set of 606 identified metabolites as previously discussed.

### Microbiome sequencing

Fecal microbiome analyses by shotgun metagenomic sequencing using the Illumina (San Diego, CA, USA) platform were performed using CosmosID (Germantown, MD, USA). Briefly, fecal DNA was extracted using the QIAGEN Dneasy PowerSoil Prokit (QIAGEN Sciences, Germantown, MD, USA) following the manufacturer’s protocol and quantified using a Qubit 4 fluorometer with the Qubit dsDNA HS Assay Kit (Thermo Fisher Scientific, Waltham, MA, USA). DNA libraries were prepared using the Nextera XT DNA Library Preparation Kit (Illumina) and IDT Unique Dual Indexes with a total DNA input of 1 ng. Per manufacturer’s protocol, genomic DNA was fragmented using a proportional amount of Illumina Nextera XT fragmentation enzyme. Unique dual indexes were added to each sample followed by 12 cycles of PCR to construct libraries. DNA libraries were purified using AMpure magnetic Beads (Beckman Coulter, Brea, CA, USA) and eluted in QIAGEN EB buffer. DNA libraries were again quantified using a Qubit 4 fluorometer and a Qubit dsDNA HS Assay Kit. Libraries were then sequenced on an Illumina HiSeq X platform with paired reads (2 × 150 bp) to a target depth of 6M reads.

### Metagenomic analysis

#### Bioinformatic processing

All sequence processing was completed using the bioBakery 3 shotgun metagenomic workflow (83), with components as described below. First, metagenomes were checked for read quality and removal of host (canine) reads. KneadData version 0.7.7 was used with default parameters to align all reads to a custom dog reference genome database based on GCA_014441545.1 (84) to remove contaminant reads from each file. This included trimming and low-quality read removal via Trimmomatic (85), first through a sliding window trim set at a window size of 4, with an average quality score of 20 for the window. Once cut, the minimum length for the remaining sequence had to be longer than 50 base pairs. The remaining mean ± SD read count per sample was 3,988,494 ± 1,941,991.

#### Taxonomic and functional profiling

High-quality reads from each metagenome were also taxonomically profiled using MetaPhlAn version 3.0 (86). For analysis, the resulting feature tables were stratified by taxonomic level (species, unless otherwise stated). Species with a minimum of 10% prevalence at 0.01% relative abundance were considered for downstream analysis. Each metagenomic sample was also functionally profiled using HUMAnN version 3.0.0 alpha 4 (87). Briefly, HUMAnN leverages the same per-species pangenomes as MetaPhlAn to create species-specific functional databases and then aligned the reads to those pangenomes again using Bowtie2 (88). Any reads remaining after this search were then translated to the corresponding amino acids and searched against UniRef90 (89) using DIAMOND (90). Once generated, HUMAnN genes were regrouped into EC numbers for analysis in RPKM (reads per kilobase of transcript per million reads mapped) units.

### Statistics and visualization

#### Statistics

Principal coordinate analysis and procrustes analysis were carried out using the “cmdscale” and “protest” functions in v2.6.4 (91), respectively. Correlations were performed and plotted using the “ggscatter” function in the ggpubr package. Pearson correlation coefficients and *P* values were added using the “stat_cor” function in the same package.

Two primary classes of statistical testing were used: omnibus and per-feature tests. The former tests whether the whole microbial community structure was significantly different across phenotypes or covariates, whereas the latter assessed this for individual microbial or metabolic features. Omnibus tests were carried out using BC distance-based PERMANOVA for the species and metabolite feature tables using the vegan v2.6.4 package (91) in R. BC distances were calculated using the “vegdist” function in the vegan package. All models were run in a univariate format (i.e., adonis[BC distance matrix ∼ x]), where x was the food or macronutrient intake (g/MBW), with 1,000 permutations. Subject ID was used as a blocking factor to set up permutation blocks for the PERMANOVA.

MaAsLin 2 v1.4.0 (92) was used to identify associations of species, enzymes (ECs), and metabolites including SCFAs with the nine macronutrients. MaAsLin 2 used a transformed generalized linear model to associate each feature iteratively with covariates of interest, here using a variance-stabilizing log transformation plus a small pseudo-count of half-the-minimum-feature value for microbial relative abundances (total sum scaling). It then modeled each microbial feature as a function of the subject’s age and adjusted the resulting *P* values for multiple hypothesis tests using BH correction. For each feature, a mixed-effect linear model was used where the fixed effect was macronutrient intake (g/MBW) and the random effect was subject (Subject ID) (i.e., lm(feature ∼ macronutrient + [1|Subject ID]).

To identify species that were differentially abundant between food groups (Fig. 4a), first, relative abundances of species were arcsine square-root transformed. Then, the mixed effect model for each species was fitted using the following formula: species ∼ food group + (1| Subject ID), with the “lmer” function from the lmerTest package. Post hoc analysis was carried out using the emmeans package in R. For each species, EMMs for food groups were calculated using the “emmeans” function, and all versus all comparisons were done using the pairs function. Species with an adjusted *P* value < 0.1 were reported as significantly differentially abundant between food groups.

#### Visualizations

Visualizations were created using functions in R packages: ggplot2 v3.4.4 (93), ggpubr v0.6.0 (94), and cowplot v1.1.3 (95).

## Data Availability

Sequences were deposited in the NCBI Sequence Read Archive under accession number PRJNA925857. Full metabolomic data from the study are available in Table S5.

## SUPPLEMENTAL MATERIAL

Supplemental figures and captions msystems.00452-24-s0001.pdf Figures S1-S10 and captions of Tables S1-S9.

Table S1 msystems.00452-24-s0002.xlsx Animal signalment.

Table S2 msystems.00452-24-s0003.xlsx Food formulations.

Table S3 msystems.00452-24-s0004.xlsx Taxonomic profiles of the 226 gut metagenomes.

Table S4 msystems.00452-24-s0005.xlsx Metabolite descriptive statistics.

Table S5 msystems.00452-24-s0006.xlsx Full metabolomics data.

Table S6 msystems.00452-24-s0007.xlsx Microbiome and metabolome BC distances.

Table S7 msystems.00452-24-s0008.xlsx Feature-wise associations with macronutrients.

Table S8 msystems.00452-24-s0009.xlsx Species that are differentially abundant between food groups.

Table S9 msystems.00452-24-s0010.xlsx Subject-wise significant associations.

ASM does not own the copyrights to Supplemental Material that may be linked to, or accessed through, an article. The authors have granted ASM a non-exclusive, world-wide license to publish the Supplemental Material files. Please contact the corresponding author directly for reuse.
