# Supplementary material for: Response of the gut microbiome and metabolome to dietary fiber in healthy dogs
Source: mSystems. 2024 Dec 23;10(1):e00452-24. doi: 10.1128/msystems.00452-24 (PMC11748496; doi:10.1128/msystems.00452-24)
Supplement: Supplemental figures and captions — Figures S1-S10 and captions of Tables S1-S9. [file msystems.00452-24-s0001.pdf]

Supplemental information for

Response of the gut microbiome and metabolome to dietary fiber in healthy dogs

Amrisha Bhosle[1,2,3], Matthew I. Jackson[4], Aaron M. Walsh[2], Eric A. Franzosa[1,2,3], Dayakar V. Badri[4]*, Curtis Huttenhower[1,2,3,5]*

[1]Infectious Disease and Microbiome Program, Broad Institute of MIT and Harvard, Cambridge, MA, United States of America
[2] Department of Biostatistics, Harvard T. H. Chan School of Public Health, Boston, MA, United States of America
[3] Harvard Chan Microbiome in Public Health Center, Harvard T. H. Chan School of Public Health, Boston, MA, United States of America
[4] Hill's Pet Nutrition, Inc., Topeka, KS, United States of America
[5] Department of Immunology and Infectious Diseases, Harvard T. H. Chan School of Public Health, Boston, MA, United States of America

*Corresponding authors: chuttenh@hsph.harvard.edu, dayakar_badri@hillspet.com

Supplemental Figures

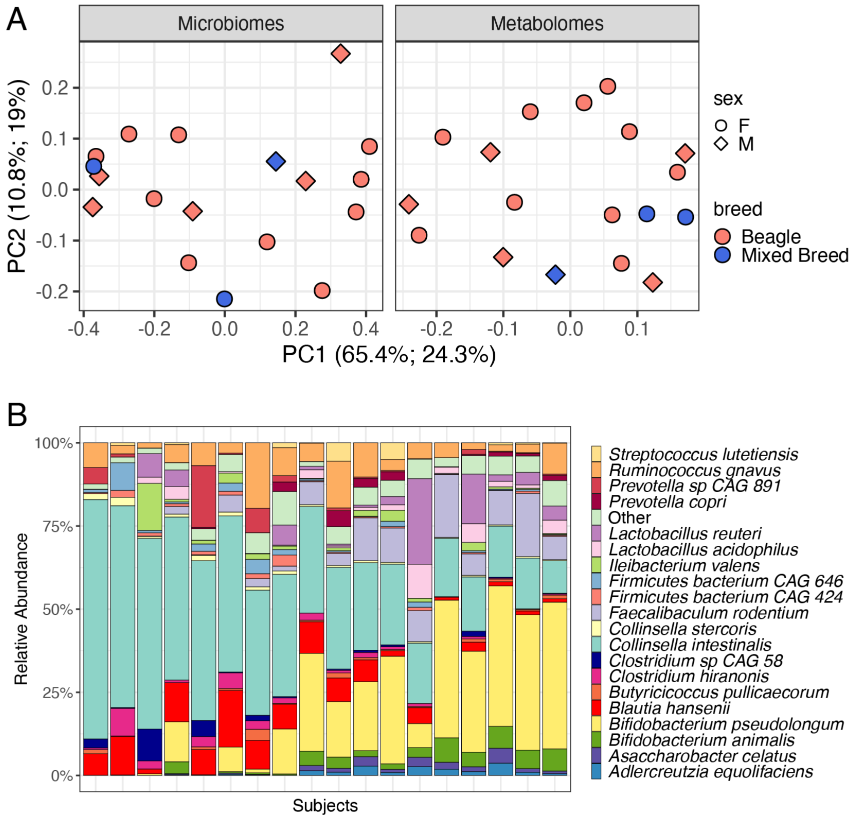

Fig. S1 (A) Bray-Curtis principal co-ordinate analysis shows no effect of breed on baseline microbiomes and metabolomes. (B) Relative abundances of microbial species in the gut microbiomes of dogs in this study in response to HSLF_Con_1 (control) food.

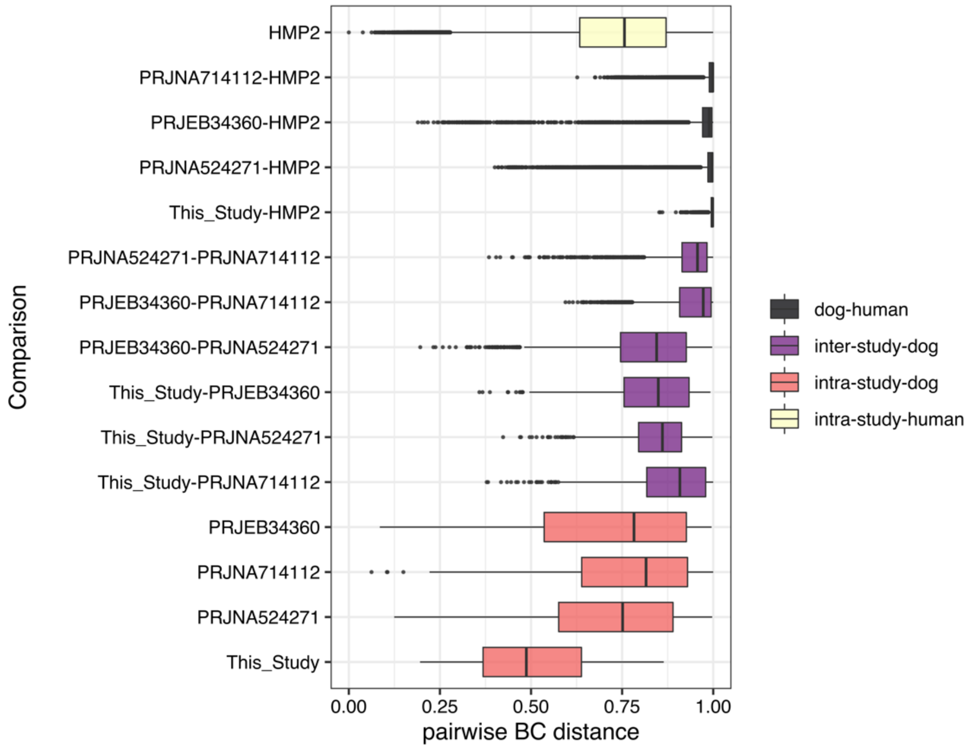

Fig. S2 Distribution of pairwise Bray-Curtis distances of comparisons of microbiomes of healthy dogs (18 baseline microbiomes) in this study, 3 public datasets of dog gut microbiomes – PRJNA324271 (N = 40; longitudinal gut microbiomes), PRJNA714112 (N = 56), and PRJEB3360 (N = 48), and healthy humans in the HMP2 cohort (N = 363; longitudinal gut microbiomes from 26 subjects). HMP2, Integrative Human Microbiome Project.

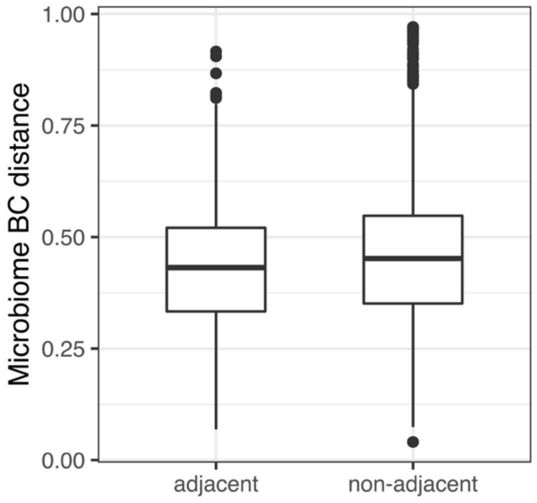

Fig. S3 Boxplots of Bray-Curtis distance of microbiomes resulting from foods fed in an adjacent or non-adjacent order. Adjacency of feeding did not have a significant effect on the similarity of microbiomes

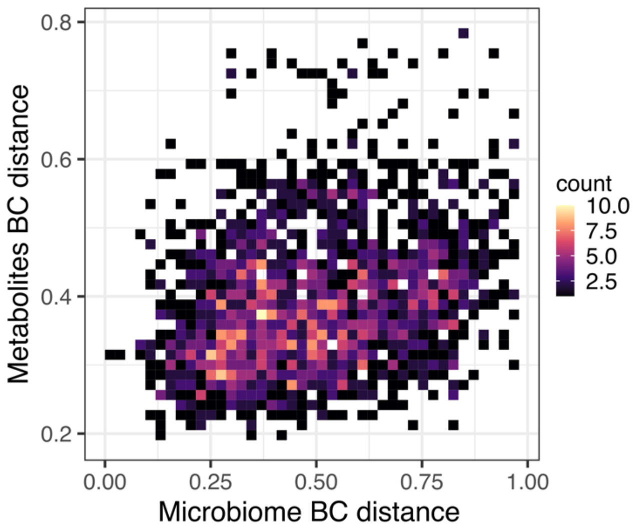

Fig. S4 Density plot showing relationships between the BC distances for the microbiomes and metabolomes for all test foods. Microbiome and metabolome BC distances are correlated ((Spearman r = 0.29, *p* < 0.0001).

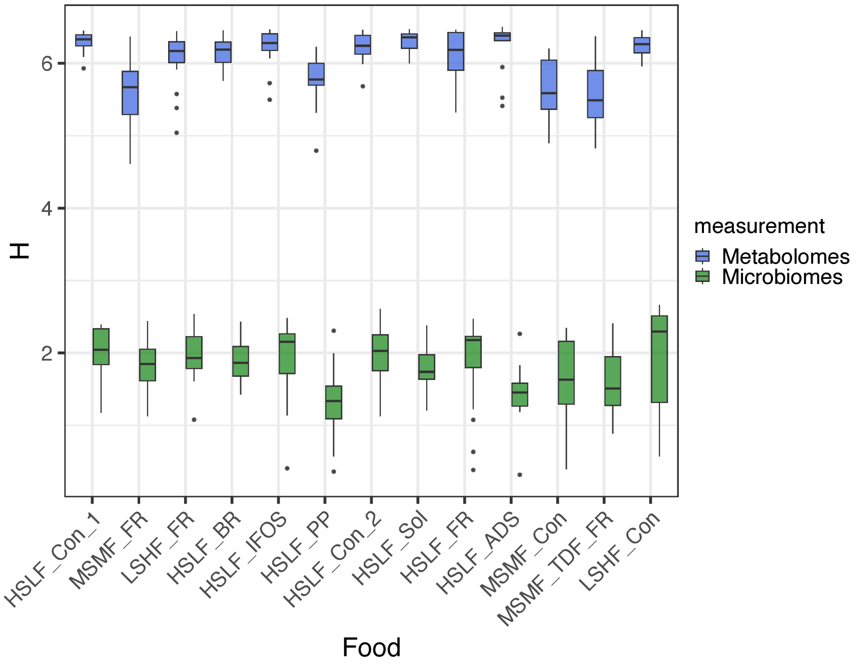

Fig. S5 Alpha diversity of the microbiomes and metabolomes reported as Shannon's diversity index (H). Lines within the boxes indicate median values. The microbiome that resulted in response to consumption of HSLF_PP was the least alpha-diverse (mean ± SD: 1.32 ± 0.49), while the one resulting from LSHF_Con (1.97 ± 0.66) was the most diverse. All of the metabolomes from consumption of the HSLF and LSHF foods had a median H > 6 except for HSLF_PP. All of the resultant metabolomes from consumption of foods in the MSMF group were less diverse (H < 6). ADS, adsorbant; BR, brewers' rice; Con, control; FR, fiber ratio; HSLF, high starch, low fiber; IFOS, inulin, fructooligosaccharides; LSHF, low starch, high fiber; MSMF, medium starch, medium fiber; PP, plant protein; Sol, high soluble fiber; TDF, total dietary fiber.

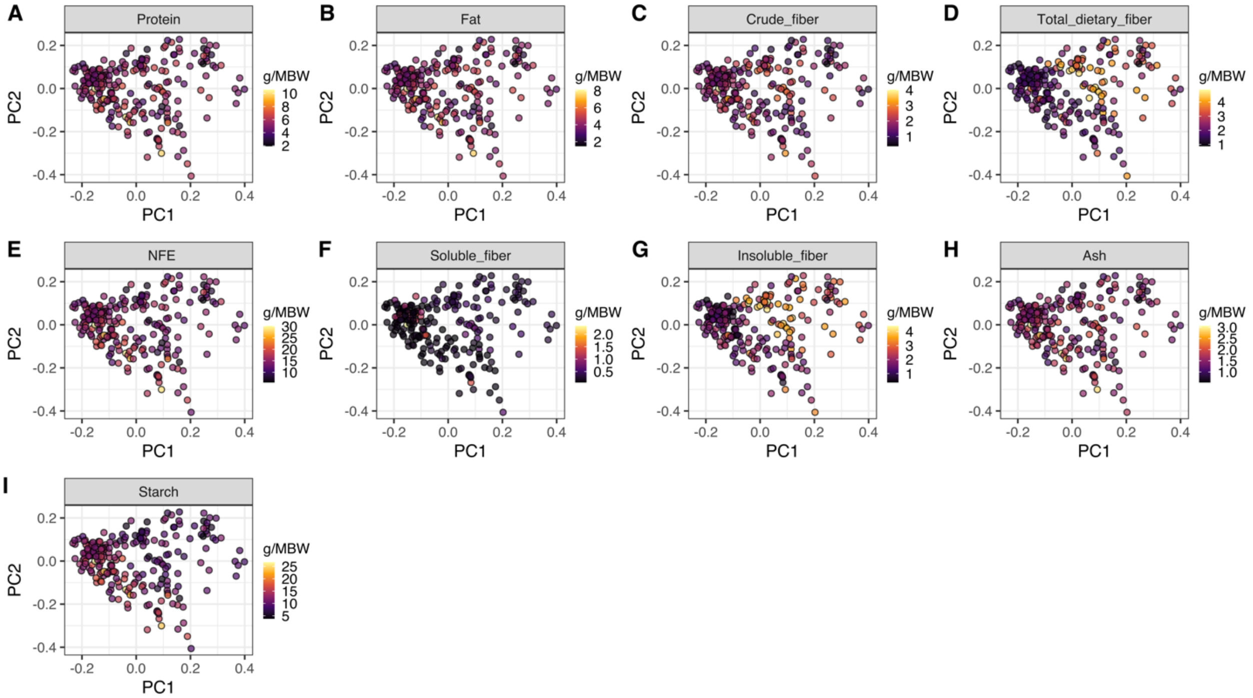

Fig. S6 Bray-Curtis distance-based ordination of metabolomes corresponding with the intake (in g/MBW) of various macronutrients in the test foods. MBW, metabolic body weight; NFE, nitrogen-free extract; PC, principal coordinate.

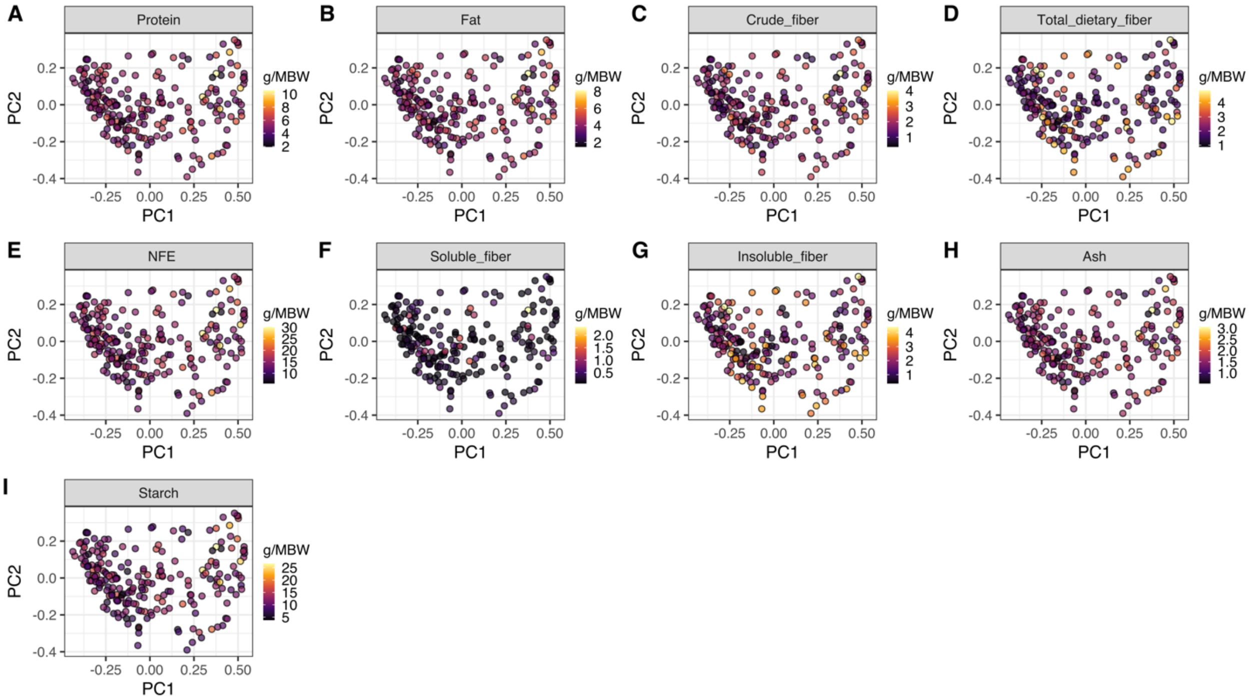

Fig. S7 Bray-Curtis distance-based ordination of microbiomes corresponding with the intake (in g/MBW) of various macronutrients in the test foods. MBW, metabolic body weight; NFE, nitrogen-free extract; PC, principal coordinate.

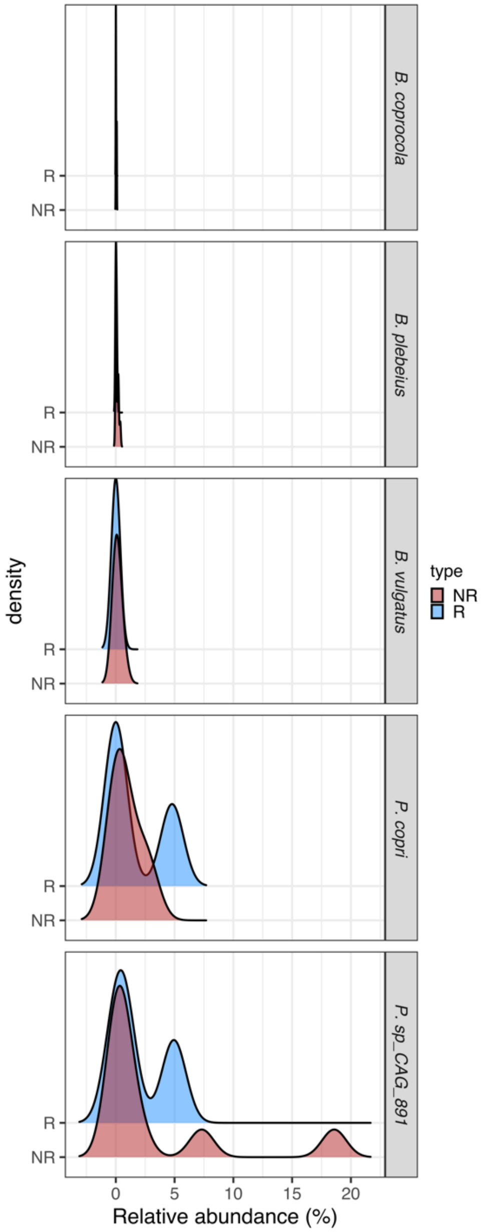

Fig. S8 Relative abundance of *Bacteroides* and *Prevotella* species in control (HSLF_Con_1) food metabolomes of responders (R) and non-responders (NR).

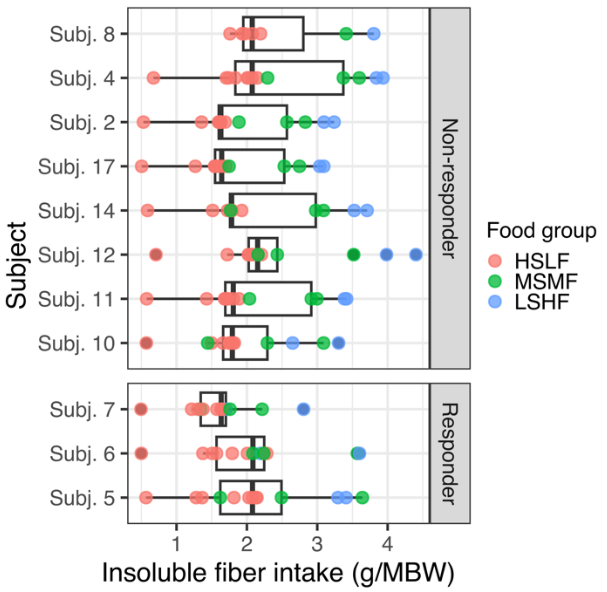

Fig. S9 Insoluble fiber intake of responders (n=3) and non-responders (n=8). HSLF, high starch, low fiber; LSHF, low starch, high fiber; MBW, metabolic body weight; MSMF, medium starch, medium fiber.

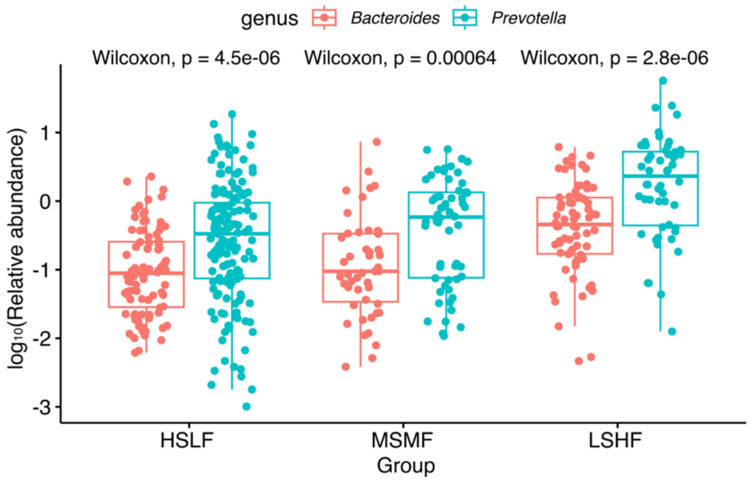

Fig. S10 Comparison of relative abundance of *Bacteroides* and *Prevotella* species in food groups. Each point represents a sample. HSLF, high starch, low fiber; LSHF, low starch, high fiber; MSMF, medium starch, medium fiber.

Supplemental Tables

Table S1 Animal signalment. Details including sex, age, and breed of dogs in the study. (XLSX)

Table S2 Formulations of the 12 foods used in the study; numbers indicate percentages in each food. Food intakes for the 18 study dogs presented as mean ± SD kcal/MBW. (XLSX)

Table S3 MetaPhlAn v 3.0 taxonomic profiles of the 226 gut metagenomes showing relative abundances at the different taxonomic levels and sample metadata. (XLSX)

Table S4 Class, subclass, and median and mean abundances of metabolites in the baseline gut metabolomes of dogs in this study. COV, coefficient of variation; HMDB ID, human metabolome database identifier. (XLSX)

Table S5 Full metabolomics data from this study. (XLSX)

Table S6 Microbiome and metabolome BC distances. (XLSX)

Table S7 Associations between individual microbial features (species, enzymes) and metabolic features (chemicals, fatty acids, dietary macronutrients) and macronutrient intake (g/MBW). Associations were identified using MaAsLin 2 [53]. EC, Enzyme Commission; NFE, nitrogen-free extract; SCFA, short-chain fatty acid. (XLSX)

Table S8 Species that are differentially abundant between food groups identified by linear mixed-effect models and comparison of EMMs. EMM, estimated marginal mean; CAG, co-abundant gene; HSLF, high starch, low fiber; LSHF, low starch, high fiber; MSMF, medium starch, medium fiber. (XLSX)

Table S9 Species (n=37) and metabolites (n=100) that retained significant association with macronutrients (insoluble fiber, soluble fiber, total dietary fiber, and starch) in each subject. (XLSX)
